# Assessment of Entrustable Professional Activities Using a Web-Based Simulation Platform During Transition to Emergency Medicine Residency: Mixed Methods Pilot Study

**DOI:** 10.2196/32356

**Published:** 2021-11-17

**Authors:** Cynthia R Peng, Kimberly A Schertzer, Holly A Caretta-Weyer, Stefanie S Sebok-Syer, William Lu, Charissa Tansomboon, Michael A Gisondi

**Affiliations:** 1 Department of Emergency Medicine Stanford University School of Medicine Palo Alto, CA United States; 2 Cornell University College of Engineering Ithaca, NY United States; 3 Stanford University Palo Alto, CA United States

**Keywords:** simulation, graduate medical education, assessment, gamification, entrustable professional activities, emergency medicine, undergraduate medical education

## Abstract

**Background:**

The 13 core entrustable professional activities (EPAs) are key competency-based learning outcomes in the transition from undergraduate to graduate medical education in the United States. Five of these EPAs (EPA2: prioritizing differentials, EPA3: recommending and interpreting tests, EPA4: entering orders and prescriptions, EPA5: documenting clinical encounters, and EPA10: recognizing urgent and emergent conditions) are uniquely suited for web-based assessment.

**Objective:**

In this pilot study, we created cases on a web-based simulation platform for the diagnostic assessment of these EPAs and examined the feasibility and acceptability of the platform.

**Methods:**

Four simulation cases underwent 3 rounds of consensus panels and pilot testing. Incoming emergency medicine interns (N=15) completed all cases. A maximum of 4 “look for” statements, which encompassed specific EPAs, were generated for each participant: (1) performing harmful or missing actions, (2) narrowing differential or wrong final diagnosis, (3) errors in documentation, and (4) lack of recognition and stabilization of urgent diagnoses. Finally, we interviewed a sample of interns (n=5) and residency leadership (n=5) and analyzed the responses using thematic analysis.

**Results:**

All participants had at least one missing critical action, and 40% (6/15) of the participants performed at least one harmful action across all 4 cases. The final diagnosis was not included in the differential diagnosis in more than half of the assessments (8/15, 54%). Other errors included selecting incorrect documentation passages (6/15, 40%) and indiscriminately applying oxygen (9/15, 60%). The interview themes included psychological safety of the interface, ability to assess learning, and fidelity of cases. The most valuable feature cited was the ability to place orders in a realistic electronic medical record interface.

**Conclusions:**

This study demonstrates the feasibility and acceptability of a web-based platform for diagnostic assessment of specific EPAs. The approach rapidly identifies potential areas of concern for incoming interns using an asynchronous format, provides feedback in a manner appreciated by residency leadership, and informs individualized learning plans.

## Introduction

In 2013, the Association of American Medical Colleges conceptualized and developed 13 activities that all incoming residents should be entrusted to perform without direct supervision on the first day of residency [1]. These 13 entrustable professional activities (EPAs) aimed to establish uniformity in skills expected of medical school graduates in the United States [2]. EPA assessment across medical schools, however, remains inconsistent [3]. Residency program directors observe significant variability in skills among incoming interns. This may result in the need to create introductory level curricula to *remediate* interns on arrival and increase faculty supervision demands in the clinical learning environment in order to bolster patient safety [4-6].

The transition from undergraduate medical education (UME) to graduate medical education (GME) continues to challenge trainees and educators, making it an important target for medical education reform [7]. The EPA framework is designed to establish a continuum from UME to GME in US-based medical education settings. As UME continues to adopt competency-based medical education, EPAs offer a complementary assessment system based on holistic, observable, and behavioral determinants of performance [8,9]. Current approaches to EPA assessment leverage existing medical school clerkships, simulation centers, or capstone programs [10,11]. Although convenient, these traditional methods of student assessment often fail to collect adequate data for competency decisions across all 13 EPAs. For example, Colbert-Getz et al [12] analyzed the content of over 400 free-text comments by physician assessors and found limited evidence supporting a student’s ability to interpret diagnostic tests (EPA3), enter orders or prescriptions (EPA4), or recognize patients requiring urgent intervention (EPA10). This assessment gap threatens the use of EPAs and calls into question whether a new approach for collecting assessment data is warranted.

One of the primary challenges to closing the EPA assessment gap is the lack of standardization across all medical schools. The EPA framework was intended to address this challenge, yet there remains significant variability in assessment methods and reporting [3]. Studies have demonstrated a trend toward using digital adjuncts for medical education [13]. The ability to use these virtual platforms has not been fully taken advantage of in assessing learners during this transition period.

The objective of this pilot study was to examine the feasibility and acceptability of asynchronous EPA assessment using a virtual platform. We report the use of this web-based interface for a selected number of EPAs in a cohort of entering emergency medicine interns during the transition between medical school and residency.

## Methods

### Study Design, Setting, and Population

This was a single-center, cross-sectional pilot study of simulation cases on a web-based EPA assessment platform using an exploratory mixed methods design, which entailed a quantitative analysis followed by a qualitative approach. We obtained a convenience sample of 15 incoming first-year interns of a 4-year postgraduate emergency medicine residency program at an academic institution. We purposively sampled these interns and program leadership for subsequent interviews. This study was approved by our institutional review board (protocol 49712).

### Conceptual Framework and Development

We used Kolb’s experiential learning model as the framework for developing our assessment [14]. The integration of active experimentation and concrete experience described by Kolb was achieved through web-based simulation [15]. Reflective observation and abstract conceptualization occurred during stakeholder interviews, as well as during the use of the assessment results to design individualized learning plans. The process of developing these learning plans is outside the scope of this study and therefore not reported.

We used the My Sim Cases web platform [16] to customize an assessment for participant skills based on 5 EPAs. We selected EPAs that are well suited for virtual assessment: EPA2: prioritize a Differential Diagnosis Following a Clinical Encounter; EPA3: Recommend and Interpret Common Diagnostic and Screening Tests; EPA4: Enter and Discuss Orders and Prescriptions; EPA5: Document a Clinical Encounter in the Patient Record; and EPA10: Recognize a Patient Requiring Urgent or Emergent Care and Initiate Evaluation and Management [17]. To assess feasibility, participants were asked to access the platform asynchronously anytime between their medical school graduation and the start of internship. Participants viewed a 5-minute tutorial that explained the interface prior to completing 4 virtual clinical cases.

A panel of 6 education assessment experts, residency program faculty, and clerkship leaders convened to design these 4 clinical cases. First, we proposed virtual cases based on chief complaints in common emergency medicine, including chest pain, shortness of breath, vomiting, and altered mental status. One author (CP) then drafted the cases and the corresponding appropriate clinical actions. In order to optimize content and internal structure evidence, the same panel reconvened to review and revise drafts thrice across several months until consensus was achieved. Then, a second panel of trainees (medical students and residents) and faculty members (clinical and nonclinical staff) provided feedback on the cases as well. These responses were cross-checked for consistency, as evidence of response process validity. From that review, a critical actions checklist and corresponding performance report was developed for each case. These reports use “look for” statements that help guide supervisors’ attention to aspects that need to be reassessed in the clinical environment or for which feedback should be provided. A “look for” statement based on expected history and physical examination techniques, missing actions, and harmful actions might read as follows: *look for incomplete or missing information in documentation*. The cases were implemented in the customized assessment platform. The web-based platform was pilot-tested by the second panel in 2 rounds, to further evaluate the cases for functionality, matching of item construct and content, optimal item phrasing, and overall quality control. Final pilot testing was performed by 6 emergency medicine faculty members with expertise in medical education.

**Figure 1:**
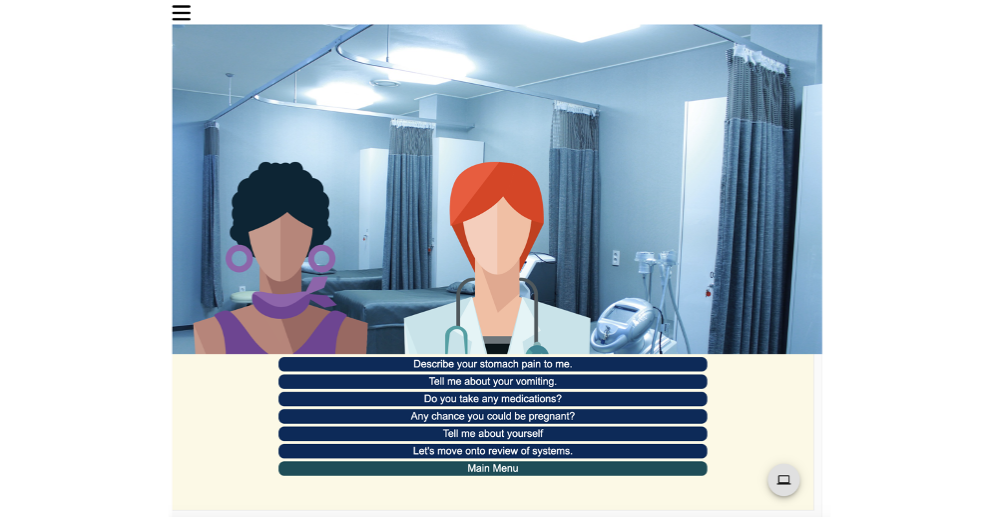
Example screenshots of web-based entrustable professional activity assessment interface.

**Figure 2:**
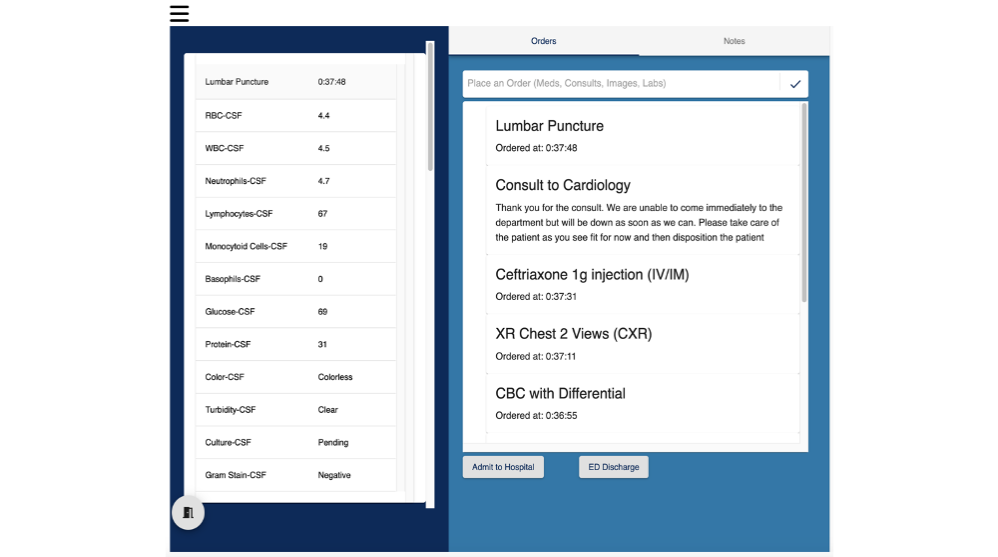
Dashboard of web-based entrustable professional activity assessment interface of electronic medical record.

The platform features 2 user interfaces that simulate the electronic medical record (EMR) and bedside evaluation (Figure 1). In the bedside interface, the participants engaged in a clinical encounter in which they clicked through questions to obtain the patient’s history, obtain additional information from prehospital providers, and request bedside clinical actions (eg, ask the nurse to display vital signs on the monitor, insert a peripheral intravenous catheter, and administer oxygen by nasal cannula). In the EMR interface, the participants placed orders for medications, imaging, labs, and requests for consultant advice (Figure 2). Participants chose the documentation of the history and physical exam using a multiple-choice prompt. They utilized an open textbox and a dropdown menu to record a differential diagnosis. The results for labs and imaging were withheld until all orders and documentation were completed. To close the encounter, the participants chose a disposition of “admit to hospital” or “discharge home” and entered a final diagnosis. A composite score report of “look for” statements was generated after completing all 4 cases; reports included between 0 and 4 “look for” statements per case with the desired score being 0.

A postassessment survey (using Qualtrics XM) captured demographic and previous training data. Following the quantitative portion of the study, we individually interviewed a subset of residents and residency program leaders about their experiences and perspectives of the assessment program and resulting data reports.

### Data Analysis

#### EPA Assessments

To evaluate feasibility of the platform, we examined participant score reports. Outcome measures included participation rates, frequency of the number of “look for” statements generated for each participant per case, frequency of the overall number of dangerous actions or missed critical actions reported per case, descriptions of the performance of each case, and overall performance per EPA. Descriptive statistics were performed using SPSS (version 27.0; IBM Corp).

#### Stakeholder Interviews

To explore acceptability of the platform, we performed qualitative interviews with key stakeholders using an interpretive phenomenological framework with thematic analysis to examine the contextual influences of their experiences [18,19]. The first author (CP) conducted individual, semistructured video conference interviews with a convenience sample of volunteer participants and the members of our residency leadership team between July and October 2020. All residency program leaders received a 15-minute presentation regarding the platform and the aggregated results prior to their interviews.

Interviews ranged from 10 to 45 minutes in length, and each was digitally recorded. Each recording was transcribed, verified, and anonymized prior to analysis. Analysis was performed by 2 independent coders (CP and CT). A codebook was established by the first coder (CT), who was blinded to the participant identities, then subsequently coded by the second coder (CP). Disagreements were discussed until consensus was reached. A Cohen kappa value was calculated using SPSS (version 27.0) to evaluate intercoder reliability [20]. Subsequently, the thematic structure of the data was analyzed based on frequency of similar content and meaning, and overall themes were determined.

#### Reflexivity

We acknowledge the potential biases that may result from previous training and experiences of our author team. Our investigators include 4 emergency physicians (CP, KS, HCW, and MAG) and 2 education researchers (SSS and CT), all of whom have experience in medical education and qualitative research methods. Two authors have served in residency leadership as emergency medicine residency program directors (MG) or associate program directors (HCW), and the other two are medical simulationists (CP and KS). All of these authors have extensive experience in training emergency medicine residents in clinical and nonclinical settings. We also had a nonclinical, but emergency medicine (EM)–focused, assessment expert (SSS) who has experience in developing several assessment innovations. Five of our authors (CP, KS, HCW, SSS, and MAG) participated in case development and the analysis meetings to ensure our individual biases were made explicit and addressed. We also sought expert panels of other EM educators who were not involved in the study design or case development for case review and pilot-testing. Interface testing also included several trainees from different post-graduate year levels.

## Results

### EPA Assessments

All 15 eligible subjects consented to participate in the study, and 13 (87%) provided demographic data. Of these, 54% (7/13) were women, with an average age of 28 (range 25-38) years. Only 85% (11/13) of the participants reviewed the tutorial about the interface, and 31% (4/13) of them reported use of a cognitive aid during the assessment (which is defined by any resource outside of the virtual case such as other websites or applications). The mean time to complete all 4 cases was 48.6 (range 16-91) minutes.

Table 1 summarizes the aggregated cohort score reports by EPA. All participants (15/15, 100%) generated the *look for* statement pertaining to the “ability to enter critical orders and avoid inappropriate or unnecessary orders,” which relates to EPA3 and EPA4. Moreover, all participants (15/15, 100%) had at least one incorrect action or order placed. The average number of missing, but not harmful, actions across all cases was 13.5 (range 8-25), and 40% (6/15) of the participants performed at least one harmful action.

Furthermore, over half (8/15, 54%) of the participants did not include the final diagnosis in their initial differential in at least one of the cases. At the conclusion of the case, 38% (3/8) input at least one incorrect final diagnosis. The participants were most successful in documentation, with only 40% (6/15) generating this “look for” statement (Table 2). The “look for” statement regarding “[the] lack of recognition of most likely urgent/emergent diagnosis and appropriate initial management” was generated most often when the learners indiscriminately applied oxygen to all cases (9/15, 60%).

Overall, participants performed the best on *Case D: Shortness of Breath* (median of 1 “look for” statement, range 1-2). For all other cases, a median of 2 “look for” statements was reported, with a higher range reported for Case B: Vomiting (range 1-4) and Case C: Altered Mental Status (range 1-4).

**Table 1:** Composite generated “look for” report based on 4 simulated cases for all learners (N=15).

Diagnostic statement	Corresponding EPA^a^	Frequency, n (%)
Look for learner ability to enter critical orders and avoid inappropriate or unnecessary orders.	EPA3: Recommend and Interpret Common Diagnostic and Screening Tests EPA4: Enter and Discuss Orders and Prescriptions	15 (100)
Look for ability to generate and prioritize a list of relevant differential diagnosis with inclusion of the most likely diagnosis.	EPA2: Prioritize Differential Diagnosis Following a Clinical Encounter	8 (54)
Look for incomplete or missing information in their documentation.	EPA5: Document Clinical Encounter in the Patient Record	6 (40)
Look for lack of recognition of most likely urgent or emergent diagnosis and appropriate initial management for stabilization.	EPA10: Recognize Patient requiring Urgent/Emergent Care and Initiate Evaluation and Management	9 (60)

^a^EPA: entrustable professional activity.

**Table 2:** summarizes the raw score reports for each case and participant. The median number of “look for” statements per participant was 2. Only 1 participant scored a perfect 0 “look for” statements for 1 case, and only 2 participants scored a 4—each for a different case.

Case description	Participant														
	1	2	3	4	5	6	7	8	9	10	11	12	13	14	15
Case A: chest pain	1	0	1	3	2	3	1	2	1	2	2	2	1	2	2
Case B: vomiting	2	1	3	2	2	2	1	2	1	2	4	2	1	2	2
Case C: altered mental status	3	1	1	2	2	2	1	2	1	4	3	2	1	2	2
Case D: shortness of breath	1	1	1	1	1	1	1	1	1	1	2	2	1	2	1
Median	1.5	1	1	2	2	2	1	2	1	2	2.5	2	1	2	2

### Stakeholder Interviews

We conducted 10 stakeholder interviews, 5 with intern residents (L1-L5) and 5 with residency leadership (L1-L5). The two coders achieved excellent interrater reliability for the resident transcripts (Cohen κ=0.92) and for the residency leadership transcripts (Cohen κ=1.00), with the unit of analysis being responses to questions.

We identified 90 unique content areas for both cohorts. We refined these to 3 major themes in relation to the acceptability of the web interface and our method of asynchronous EPA assessment (Table 3). These themes included (1) psychological safety, (2) assessment for learning, and (3) value of interface fidelity. Topics most commonly discussed by the participants pertained to psychological safety, user experience, and usefulness of formative feedback, whereas the focus of the faculty interviews was individualized learning and potential uses of assessment results.

**Table 3:** Themes and representative quotes from stakeholder interviews.

Theme	Trainee	Residency leader
Psychological safety	“It's really odd for someone to tell someone critical feedback or give them bad criticism in front of your other peers, because you're going to leave the room and [then] hang out together. And people are just more hesitant to [be together].”	“I want them to see this as an opportunity for growth, and I worry that if we have different cohorts, they're going to feel singled out from their peers...psychological safety is key to actually fostering growth, which is key to individualized learning. So, if we take away their sense of belonging, all of a sudden we've jeopardized the success of individualized learning.”
Assessment for learning	“It can be used as a way to...judge if [the learner] has tendencies to, say, order X, Y, and Z labs when maybe you should’ve [done] this first.”	“This tells us areas of topics that we need to highlight more and cover more in intern orientation and be more deliberate about it.”
Value of interface fidelity	Positive: “There was an interesting variety [of cases] and that they were fairly bread and butter EM. And that the interface worked fairly well and let me go through the correct order of operations that I would typically do in ED as far as assessing the patient and then getting studies and working on my diagnosis and treatment plan.” Negative: “I did feel that sometimes the diagnoses were restrictive, or some of the things that we could do were a little restrictive.”	“The ordering practices and the items that they choose to order or not order are probably the most helpful.... the students haven't had to actually put in orders until they reach residency.”
Cohen Kappa	0.92	1.00

#### Psychological Safety

Most participants reported that the web-based assessment platform had potential to obtain individualized feedback while maintaining psychological safety. The trainees reported that privacy is not always prioritized when they receive in-person feedback. One participant (L2) noted “[feeling] odd…[hearing] bad criticism in front of...other peers.” In contrast, they felt that security and privacy of our web-based interface could offer targeted, individualized feedback without the need for normative comparisons to peers.

Although diagnostic assessment can group individuals according to different strengths and weaknesses, one faculty member (L1) stressed that “psychological safety is key to fostering growth, which is key to individualized learning.” The residency leaders identified individualized learning as the ultimate goal for use of this platform. Safety in the score reports was also emphasized by faculty, and they suggested several instructions for how to interpret the reports. Score reports should ensure a trainee’s “sense of belonging” (L1) in their new program, irrespective of performance. Another faculty member (L3) suggested deliberate statements such as “everybody will have areas they need to develop” in order to normalize the results of the assessment.

#### Assessment for Learning

Trainees found that immediate feedback provided by the interface was the most important feature to leverage for learning. These learners (L1-L5) cited that the “biggest difference” in learning was having “really specific feedback [...] in real time.” This assessment for learning was more important to the participants than the assessment of the EPAs themselves. In addition, one participant (L1) suggested adding more cases to the platform to allow exploration of a range of diagnoses prior to their residency start date, from “common chief complaints...to rare ones.” They also suggested that the assessment interface could become an adjunct to the residency curriculum if paired with specific clinical rotations.

Faculty participants agreed that the ability to “assess where [the interns] are...from day one” (L1) would offer valuable information needed to begin tailored training. They believed that the assessment would allow for just-in-time curriculum redesign of residency orientation topics based on the cohort performance. Aside from “identifying areas [of deficiency],” the residency leaders (L2 and L4) reported that the information could be used for “remediation” of individuals who might otherwise make similar patient care errors early in training.

#### Value of Interface Fidelity

Overall, the trainees found the simulation to be representative of their clinical experiences. One intern (L3) noted that it “really made [him] think” about the “order of operations” in a case and by “forc[ing him] to go step by step.” Furthermore, they agreed that the interface helped them (L2) “understand the workflow, the efficiencies, the logistics of dealing with a patient,” leading them to think about “how to be efficient” (L2 and L4).

Our current interface has certain limitations. One trainee (L3) cited that the experience of inputting the diagnoses felt “restrictive” due to prompts and preset options and that participants would have preferred to “[free] type in answers.” Others (L2) noted that the interface required many nonessential navigation clicks that “weren’t really changing management.” These issues represent important threats to fidelity.

The residency leaders valued the use of “look for” statements regarding missing and incorrect actions, in contrast to a numerically scored option. “Look for” statements were reported as representative of the kind of feedback one might expect in the clinical unit. They cited the interface as being particularly beneficial for evaluating the ordering practices of the trainee. One participant (L5) noted that the ability to see “the critical actions and harmful actions component of the online platform […] the most valuable component.”

## Discussion

### Principal Findings

The use of a web-based simulation platform for EPA assessment is feasible and acceptable to key stakeholders (ie, residents and program leadership). EPA assessment using simulated cases that were customized to common emergency medicine chief complaints may have increased fidelity for incoming interns and relevance of the score reports to their program directors. Trainees found that asynchronous, individual testing provided psychological safety, and residency directors believed that the score reports could guide the development of individualized learning plans early in residency training. Psychological safety and targeted, individualized feedback are desirable outcomes consistent with other EPA-based studies [21-23].

Importantly, no single participant was competent across all 5 EPAs. These findings are somewhat troubling yet promising that our virtual assessment detected such information. Our study also highlights the need for medical schools to better use the EPA framework to guide curriculum decisions and assure the quality of their graduates upon summative entrustment for preparedness to enter into residency training. These findings are neither surprising nor novel, and they are consistent with other EPA literature to date [3,24,25].

Acceptability of this assessment approach was strengthened by the use of “look for” statements rather than numerical scores. Rigorous EPA assessment by medical schools would use multisource feedback and standardized testing to achieve defensible decisions about student competence. “Look for” statements mirror high-quality clinical feedback that is familiar to most students. It also operationalizes the EPA assessment for program directors beyond a construct of *competent versus incompetent* that might be offered in a summative report at the end of medical school. Moreover, these statements provide trainees and educators with understandable and achievable learning goals and align better with the culture of feedback rather than a punitive approach to learning, while simultaneously highlighting significant educational gaps.

Another important factor to consider with a customized design is the physician order entry interface that could simulate the EMR used in the local clinical environment. Consistency between the simulated platform and the local EMR could result in early adoption of systems at the sponsor institution and reduce cognitive load once interns begin clinical rotations. Outcomes of EMR training have shown a reduction in self-reported medical errors, and similar benefits could be observed with this assessment for learning during orientation [17,26].

### Limitations

The limitations of this study include a small sample size, a single institution pilot, a single specialty cohort, implicit biases noted in the study methods, and the use of a convenience sample of stakeholders. Although all residency directors or associate directors in our department were interviewed, we sought volunteers from the trainee cohort; these could be individuals who may have had a favorable opinion of the pilot study and thus offered to volunteer, which could affect their responses. Similarly, the stakeholders may have inferred that the interviewer had a favorable opinion of the project and therefore softened any potentially negative responses. Our cases are aligned with our specialty to increase response process validity; thus, further testing is required to explore generalization of these results across students entering any field. Finally, this assessment is meant to be formative, not summative; we cannot fully assess student competence for any EPA with a single test and absence of multisource data. Therefore, the results of this assessment are best used by trainees and residency directors, not medical schools seeking a single examination of EPA competence.

Future study of our platform will include the logical expansion of testing to all medical school graduates to ensure this observation remains consistent. Customization of EPA assessments using specialty-relevant cases is also desirable for fidelity; as such, specialty boards might be the logical third parties to oversee development of such interfaces. This would bookend a resident’s interaction with their future boards, with incoming assessment and certification exams at either end. Further investigation should also include a longitudinal evaluation of clinical learning outcomes at various intervals during residency training. Finally, it is critical to examine the design of individualized learning plans based on assessment results. Although the benefits of individualized learning and development plans have been previously demonstrated, their implementation has been difficult in the absence of pragmatic guidelines [2,27].

### Conclusions

Asynchronous, individual EPA assessment using this web-based platform is feasible and acceptable to key stakeholders. This offers a psychologically safe and yet practice-relevant way to diagnostically assess incoming interns and will assist with transitions to residency.

## Appendix

Multimedia Appendix 1 Interview questions for qualitative analysis.

## Abbreviations

EMR: electronic medical record
EPA: entrustable professional activities
GME: graduate medical education
UME: undergraduate medical education
