# Supplementary material for: Assessment of Entrustable Professional Activities Using a Web-Based Simulation Platform During Transition to Emergency Medicine Residency: Mixed Methods Pilot Study
Source: JMIR Med Educ. 2021 Nov 17;7(4):e32356. doi: 10.2196/32356 (PMC8663509; doi:10.2196/32356)
Supplement: Multimedia Appendix 1 [file mededu_v7i4e32356_app1.docx]

**Multimedia Appendix 1.** Interview questions for qualitative analysis.

|  | **RLT Questions** | **Intern Questions** |
| --- | --- | --- |
| **1.** | What is your role in residency education? What has been your experience in residency leadership and your experience with individualized learning? | Based on your medical training experience so far, what has your experience been like with individualized feedback? |
| **2.** | In general, what are your thoughts regarding incoming interns and their preparedness for providing clinical care? | What was your experience in using this online interface? |
| **3.** | How do you envision an online interface being used for individualized learning? | How would you like to see this online platform being used? |
| **4.** | What were your expectations for this diagnostic assessment and What did you anticipate for the results? | To what extent do you think this online platform can be used for teaching and feedback? |
| **5.** | What part of this platform is the most valuable? How would you envision your program using the reports generated from this assessment? | Do you think there would be an impact on your performance if this was being used for feedback? If so, how so? |
